# Endoplasmic Reticulum (ER) Stress-Generated Extracellular Vesicles (Microparticles) Self-Perpetuate ER Stress and Mediate Endothelial Cell Dysfunction Independently of Cell Survival

**DOI:** 10.3389/fcvm.2020.584791

**Published:** 2020-12-10

**Authors:** Aisha Osman, Heba El-Gamal, Mazhar Pasha, Asad Zeidan, Hesham M. Korashy, Shahenda S. Abdelsalam, Maram Hasan, Tarek Benameur, Abdelali Agouni

**Affiliations:** ^1^Department of Pharmaceutical Sciences, College of Pharmacy, QU Health, Qatar University, Doha, Qatar; ^2^Department of Basic Sciences, College of Medicine, QU Health, Qatar University, Doha, Qatar; ^3^College of Medicine, King Faisal University, Al-Ahsa, Saudi Arabia

**Keywords:** extracellular vesicles (EVs), microparticles (MPs), endothelial function (dysfunction), apoptosis, cardiovascular disease, ER stress

## Abstract

Circulating extracellular vesicles (EVs) are recognized as biomarkers and effectors of endothelial dysfunction, the initiating step of cardiovascular abnormalities. Among these EVs, microparticles (MPs) are vesicles directly released from the cytoplasmic membrane of activated cells. MPs were shown to induce endothelial dysfunction through the activation of endoplasmic reticulum (ER) stress. However, it is not known whether ER stress can lead to MPs release from endothelial cells and what biological messages are carried by these MPs. Therefore, we aimed to assess the impact of ER stress on MPs shedding from endothelial cells, and to investigate their effects on endothelial cell function. EA.hy926 endothelial cells or human umbilical vein endothelial cells (HUVECs) were treated for 24 h with ER stress inducers, thapsigargin or dithiothreitol (DTT), in the presence or absence of 4-Phenylbutyric acid (PBA), a chemical chaperone to inhibit ER stress. Then, MPs were isolated and used to treat cells (10–20 μg/mL) for 24–48 h before assessing ER stress response, angiogenic capacity, nitric oxide (NO) release, autophagy and apoptosis. ER stress (thapsigargin or DDT)-generated MPs did not differ quantitatively from controls; however, they carried deleterious messages for endothelial function. Exposure of endothelial cells to ER stress-generated MPs increased mRNA and protein expression of key ER stress markers, indicating a vicious circle activation of ER stress. ER stress (thapsigargin)-generated MPs impaired the angiogenic capacity of HUVECs and reduced NO release, indicating an impaired endothelial function. While ER stress (thapsigargin)-generated MPs altered the release of inflammatory cytokines, they did not, however, affect autophagy or apoptosis in HUVECs. This work enhances the general understanding of the deleterious effects carried out by MPs in medical conditions where ER stress is sustainably activated such as diabetes and metabolic syndrome.

## Introduction

Maintaining optimal vascular homeostasis balance is very critical for the integrity of the cardiovascular tree to prevent the development of cardiovascular co-morbidities particularly prevalent with metabolic diseases such as obesity and diabetes. Achieving balance between relaxing and contracting factors, and between antithrombotic and prothrombotic factors is important for the normal function of the vascular system. These functions are maintained by the endothelium, a thin layer of endothelial cells that line the entire vasculature. Perturbation of endothelial functions in diseases such as diabetes and insulin resistance states disturb this balance leading to a state of endothelial dysfunction, the early step in the process of atherogenesis, resulting in cardiovascular complications in patients with cardiovascular risk factors such as obesity, diabetes and metabolic syndrome (1). Features of endothelial dysfunction in include: (i) impairment of vascular function indicated by reduced nitric oxide (NO) bioavailability, impaired endothelium-dependent smooth muscle relaxation and perturbated angiogenesis; (ii) induction of vascular inflammation marked by increased production of inflammatory mediators and adhesion molecules; and (iii) activation of a prothrombotic state by increasing the production of procoagulant factors and enhancing platelet aggregation.

The mechanisms underlying the development of endothelial dysfunction are not yet fully understood. However, in recent decade, circulating extracellular vesicles (EVs) have emerged as useful biomarkers, predictors and effectors of endothelial dysfunction that is known to be associated with metabolic abnormalities including obesity, diabetes and metabolic syndrome. EVs are typically classified in the literature according to their size and mechanism of biogenesis and release from the cell. The main EVs subtypes are exosomes, apoptotic bodies and microparticles (MPs). Exosomes range in size from 40 to 120 nm and are released from cells by fusion of multivesicular bodies (MVBs) with cell membrane and then MVBs release exosomes to the extracellular environment by exocytosis (2). While apoptotic bodies (size > 1,000 nm) and MPs (size 100–1,000 nm) appear to have similar mechanisms of release which is outward blebbing and shedding of vesicles directly from the plasma membrane of apoptotic or activated cells, respectively (2, 3).

EVs levels, particularly large-sized EVs (MPs) originating from endothelial cells, were found to be altered in patients with metabolic and cardiovascular co-morbidities. Esposito et al. (4) have found CD31+/CD42b– endothelial MPs (EMPs) and CD31+/CD42b+ platelet MPs (PMPs) to be significantly elevated in obese women with concomitant reduction of endothelium-dependent flow mediated vasodilation. EMPs were reported as independent predictors of endothelial dysfunction (4). EMPs (5), and circulating MPs from diabetic patients (6), were found to be deleterious for endothelial cell function. EMPs were reported to be elevated in diabetic patients compared to healthy controls (7). Furthermore, a significant positive correlation between EMPs and glycated hemoglobin (HbA1c) levels was observed, and EMPs were identified as independent predictors of endothelial dysfunction (reduced flow-mediated dilation of brachial artery and elevated brachial ankle pulse wave velocity) in diabetic patients (7). Feng et al. (8) and Ishida et al. (9) showed significantly elevated levels of circulating MPs from endothelial origin in streptozotocin-induced diabetic rats, compared to non-diabetic rats (8, 9).

EVs are key molecular effectors in endothelial dysfunction. In metabolic syndrome patients, endothelial, procoagulant, platelet, and erythrocyte MPs were significantly elevated compared to healthy individuals (10, 11). Agouni et al. (10) reported that EVs derived from metabolic syndrome patients were able to induce endothelial dysfunction both *in vitro* and *in vivo*. Treatment of endothelial cells with metabolic syndrome MPs decreased NO production and increased the phosphorylation of endothelial NO synthase (eNOS) at the inhibitory site (Thr495), while injection of these MPs into mice attenuated vasodilation in response to acetylcholine in aortas (10) and caused a vascular hypo-reactivity (12). However, the molecular and cellular mechanisms underpinning the deleterious actions of MPs on endothelial function are still poorly understood.

In recent years, the endoplasmic reticulum (ER) stress response has been identified as a strong molecular bridging link between insulin resistance, inflammation and endothelial dysfunction. The ER is the site for posttranslational folding and synthesis of secretory and transmembrane proteins. Upon various physiological or pathological disturbances that increase protein demand, the accumulation of misfolded or unfolded proteins can occur within the ER lumen, leading to the activation of an adaptive signaling cascade called unfolded protein response (UPR). The UPR is activated to attenuate protein synthesis, increase protein folding capacity and promote degradation of irreversibly misfolded proteins in an attempt to restore ER homeostasis (13). If ER homeostasis is not restored, ER stress response ensues which triggers signaling pathways involved in inflammation and cell apoptosis. Three transmembrane sensors located at the ER membrane: protein kinase R (PKR)-like ER kinase (PERK), inositol-requiring enzyme (IRE)-1α and activating transcription factor (ATF)-6, are activated following their dissociation from the major ER chaperone: immunoglobulin binding protein also known as glucose-regulated protein 78 (BiP/GRP78). BiP becomes thus available in the ER lumen to bind to and correct the misfolded and unfolded polypeptide chains; while the activated transmembrane sensors are involved in UPR downstream signaling pathways (13, 14). Collectively, UPR is a defensive mechanism activated to restore the proper function of the ER. However, upon its prolonged activation, a state of ER stress takes place. ER stress response by itself can result in endothelial dysfunction through various underlying molecular mechanisms including apoptosis, cellular inflammation and oxidative stress (15, 16). ER stress has been shown to be involved in endothelial dysfunction in models of diabetes and insulin resistance (15). Moreover, ER stress is linked to atherosclerosis as it was found to be activated in all stages of atherosclerosis (17). ER stress activation is closely associated with cell death which contributes to endothelial dysfunction (16, 18). ER stress-mediated cell death is linked to the activation of c-Jun N terminal kinase (JNK) and subsequent production of reactive oxygen species and activation of the autophagy (19). More recently, Safiedeen et al. (20) have shown a role for the activation of ER stress in endothelial dysfunction induced by MPs. Exposure of human aortic endothelial cells to MPs derived from metabolic syndrome patients or apoptotic T-lymphocytes enhanced the expression of several markers associated with the three arms of ER stress (PERK, IRE-1α, and ATF-6) and reduced NO bioavailability. Furthermore, MPs impaired endothelium-dependent vasodilation *in vivo* in mice. All of these actions in cultured cells and mice were reversed in the presence of tauroursodeoxycholic acid (TUDCA), a chemical chaperone known to inhibit ER stress, indicating the strong involvement of ER stress in MP-induced endothelial dysfunction (20).

Although evidence is available about the involvement of ER stress in endothelial cell dysfunction mediated by MPs (20), it is still not known whether the activation of ER stress response is a cause or a consequence of MPs generation. Also, the effects mediated by ER stress-generated MPs on endothelial cell function still need to be investigated. Therefore, the aim of this study was to assess the impact of ER stress activation on MP shedding from endothelial cells, and to investigate their effects on the activation of ER stress itself and endothelial function with special focus on angiogenic capacity, NO release and possible implication of ER stress-mediated cell death and autophagy in the process.

## Materials and Methods

All experimental work was conducted in line with Qatar University Institutional Biohazard Committee (IBC) policies and guidelines.

### Cell Culture and Maintenance

EA.hy926 endothelial cells (ATCC® CRL-2922™, Manassas, USA) were cultured in high glucose (25 mM) Dulbecco's Modified Eagle's Medium (DMEM) (Pan biotech, Aidenbach, Germany) supplemented with 10% fetal bovine serum (FBS; Gibco, Carlsbad, USA), 1% penicillin/ streptomycin (P/S; Gibco), 1% L-glutamine (Gibco) and 1% sodium pyruvate (Gibco). Cells were incubated at 37 and 5% Carbon dioxide (CO_2_) in humidified conditions.

Primary Umbilical Vein Endothelial Cells; Normal, Human (HUVECs) (ATCC® PCS100010™) were cultured in Medium 200 (Gibco) supplemented with 2% Low Serum Growth Supplement (LSGS; Gibco) and 1% P/S. Cells were incubated at 37 and 5% Carbon dioxide (CO_2_) in humidified conditions. HUVECs were used in experiments up to passage 6.

### Cell Treatments

To pharmacologically induce ER stress, cells were either incubated with thapsigargin (TG) (300 nM, 24 h) (ThermoScientific, Waltham, USA), or Dithiothreitol (DTT) (Sigma-Aldrich, Hamburg, Germany) (2 mM, 24 h). The concentrations used in this study for TG and DTT were previously shown to induce ER stress in endothelial cells (18, 21). TG is an inhibitor of sarcoplasmic reticulum/endoplasmic reticulum Ca^+2^-ATPase (SERCA); SERCA allows the transfer of Ca^+2^ from the cytoplasm to the lumen of the ER. By inhibiting SERCA with TG, the ER will be depleted from Ca^+2^ along with increasing the cytoplasmic Ca^+2^ levels. Ca^+2^ depletion results in losing the activity of Ca^+2^-dependent chaperones that fix the misfolded and unfolded proteins (22). DTT is a reducing agent that works by reducing disulfide bonds of proteins, thus contributing to ER stress and activation of UPR. To harness the role of ER stress, a chemical chaperone was used to increase ER homeostasis and block ER stress, 4-Phenylbutyric Acid (PBA) (10 mM; Sigma-Aldrich). PBA, at this concentration, was shown to alleviate pharmacologically induced ER stress in endothelial cells (18). For MPs treatments, naïve EA.hy926 and HUVECs cells were stimulated with generated MPs from respective cell line at the concentrations of 10 or 20 μg/ml of medium for 24 or 48 h. Concentrations of MPs used were based on previously published reports (6, 23).

### MPs Isolation and Quantification

MPs were isolated from culture media of treated cells (TG or DTT in the presence or absence of PBA) by serial centrifugations at 21,000 × *g* for 45 min at 4°C as described previously by us (23–25). Briefly, first, culture media were collected, and centrifuged at 1,500 × *g* for 20 min at room temperature to remove cell debris. Second, supernatants were aliquoted in Eppendorf tubes after discarding pellets and then centrifuged at 21,000 × *g* for 45 min at 4°C. Third, the supernatants were discarded, and pellets pooled from all tubes from the same treatment condition and transferred to 1 single tube, and then suspended in 1 ml of sterile and cold Phosphate Buffer Saline (PBS). Then, the suspended pellet was washed by two serial centrifugations at 21,000 × *g* for 45 min at 4°C with PBS changed each time to ensure no traces of medium are left. Finally, the supernatant was discarded and the pellet (MPs) was suspended in 300 μl of PBS and stored at 4°C until subsequent use (storage duration did not exceed 1 month for each batch). MPs were quantified indirectly by measuring the total protein content using Bicinchoninic acid (BCA) protein assay (ThermoScientific, Waltham, USA) according to the manufacturer's recommendations using a bovine serum albumin (BSA) standard curve. Each set of experiments was done using at least three different batches of MPs preparations.

### Western Blot Analysis

Following treatments, cells were washed once in PBS to remove left-over medium, and then whole-protein lysates were extracted in cold Radioimmunoprecipitation Assay (RIPA) lysis buffer [0.5 M Tris pH 6.8, 20% Sodium dodecyl sulfate (SDS) and a cocktail of protease and phosphatase inhibitors]. Protein concentrations were determined using the BCA method (ThermoScientific, Waltham, USA). Equal amounts of proteins (10–20 μg) were resolved on SDS-PAGE gels (8–12% based on the molecular weights of targets). After transfer and blocking for 1 h in tris-buffered saline (TBS) and 0.1% of Tween 20 (Sigma-Aldrich, Hamburg, Germany) (T-TBS) and 5% dry milk, the blots were washed in T-TBS and incubated overnight with respective primary antibodies. Immunoblotting was done using primary antibodies against: BiP, p-p42/44 mitogen-activated protein kinase (MAPK) (Thr202/204), p42/44 MAPK, p-c-JUN (Ser73), p-p38 (Thr180), autophagy marker light chain 3 (LC-3 I/II) (1:1,000) (Cell Signaling Technology, Danvers, USA), or mouse anti-β-actin (1:5,000) (Santa Cruz Biotechnology, Dallas, USA). After washing in T-TBS, membranes were incubated with appropriate secondary antibodies (horseradish peroxidase-conjugated) (1:10,000) and chemiluminescence signal was visualized using Enhanced chemiluminescence (ECL) reagent (Abcam, Cambridge, UK) using FluorChem M imaging system (Protein Simple, San Jose, USA) and protein band intensities were analyzed using AlphaView software (Protein Simple).

### Total RNA Isolation and Gene Expression Analysis

Following treatments, cells were washed in PBS (ThermoScientific, Waltham, USA), total RNA was extracted using the innuPREP RNA Mini kit (Analytikjena, Berlin, Germany) by following the supplier recommendations. RNA concentration and quality preparations were determined using a NanoDrop 2000 (ThermoScientific). cDNA synthesis was performed in of total RNA (500 ng) using the RevertAid reverse transcription kit (ThermoScientific) and an oligo(dT)_12−18_ primer according to the supplier's instructions. Target genes were then amplified using GoTaq qPCR Master Mix (Promega, Madison, USA) in Applied Biosystems 7500 fast Real-Time PCR System (ThermoScientific).

All experiments were performed independently with at least three biological repeats and technical replicates. Data were analyzed by the comparative Ct method, and expressed as fold change relative to control group. The pairs of primers for human target genes were sourced from Primer bank and synthesized by Sigma-Aldrich (Hamburg, Germany). Sequences of human primers used in the study are summarized in Table 1.

**Table 1:** List of human primers used for qPCR analysis.

**Target Gene**	**Forward Primer**	**Reverse Primer**
*β-actin*	CATGTACGTTGCTATCCAGGC	CTCCTTAATGTCACGCACGAT
*BiP*	CATCACGCCGTCCTATGTCG	CGTCAAAGACCGTGTTCTCG
*CHOP*	GAACGGCTCAAGCAGGAAATC	TTCACCATTCGGTCAATCAGAG
*ATF-4*	CCCTTCACCTTCTTACAACCTC	TGCCCAGCTCTAAACTAAAGGA
*GRP94*	GCTGACGATGAAGTTGATGTGG	CATCCGTCCTTGATCCTTCTCTA
*TRIB3*	AAGCGGTTGGAGTTGGATGAC	CACGATCTGGAGCAGTAGGTG
*ATG-3*	CCAACATGGCAATGGGCTAC	ACCGCCAGCATCAGTTTTGG
*Beclin-1*	TGAGGGATGGAAGGGTCTAAG	GCCTGGGCTGTGGTAAGTAATC
*IL-10*	ACTTTAAGGGTTACCTGGGTTGC	TCACATGCGCCTTGATGTCTG
*eNOS*	TGATGGCGAAGCGAGTGAAG	ACTCATCCATACACAGGACCC
*VEGF-A*	AGGGCAGAATCATCACGAAGT	AGGGTCTCGATTGGATGGCA
*FGF-2*	AGAAGAGCGACCCTCACATCA	CGGTTAGCACACACTCCTTTG

### Assessment of Apoptosis

Apoptosis in HUVECs treated with MPs (20 μg/ml for 48 h) generated from untreated HUVECs (CTL MPs) or cells treated with TG, TG + PBA or PBA, was assessed using Tali® apoptosis kit (ThermoScientific, Waltham, USA) according to the manufacturer's instructions. After treatments, culture medium was collected from the 6-well plates and transferred into 15-ml tubes. Then, cells were detached with trypsin and transferred to respective 15-ml tubes containing the old media. Media was then centrifuged at 1,500 × *g* for 5 min at 4°C, and the pellet was resuspended in 100 μl 1X Annexin Binding Buffer (ABB). Then, 5 μl of Annexin V solution were mixed to each sample and incubated at room temperature for 20 min in the dark. Samples were centrifuged again, and the pellet was resuspended in 100 μl of fresh 1X ABB. Lastly, 1 μl of propidium iodide (100 μg/ml) was added, and samples were incubated for 10 min in the dark. The samples were analyzed by loading the stained cells (25 μl) into the Tali Cellular Analysis Slides (ThermoScientific) and imaging using Tali Image-based Cytometer (ThermoScientific) following the kit protocol. The analysis included the percentages of live cells, dead cells and apoptotic cells in each cell preparation.

### Endothelial Cell Tube-Like Structures Formation Assay

*In vitro* angiogenesis assay using Geltrex™ Matrigel matrix (ThermoScientific, Waltham, USA) was followed to assess the ability of endothelial cells to form tube-like structures, an indication of their angiogenic capacity. HUVECs were seeded into 6-well plates with a seeding density of 150,000 cells/well. Next day, cells were treated with MPs (20 μg/ml) derived from untreated HUVECs (CTL MPs) and those treated with TG, TG + PBA, or PBA. Untreated cells (no MPs treatment) were used as controls. Cells were left to incubate for 48 h. Geltrex™ (ThermoScientific) was thawed in the refrigerator (4°C) overnight before use. On the day of the experiment, Geltrex™ (100 μl) was added into 48-well plates then the plate was shacked gently for a uniform layer of the gel. The gel-coated wells were incubated at 37°C for 30 min until the gel solidified. Meanwhile, treated cells were washed with PBS and detached with trypsin. The cell suspension was then centrifuged at 1,500 × *g* for 5 min. The pellet was resuspended in 100 μl of medium and counted using hemocytometer. Cells were then loaded into gel-coated wells with a seeding density of 50,000 cells/well and cell suspension volume of 100 μl. The plate was incubated at 37°C and 5% CO_2_ for 4 h. Following this, the formed tube-like structures were visualized by the phase contrast inverted microscope and images were taken (Optika, Ponteranica, Italy). Semi-quantitative image analysis was performed using WimTube software from Wimasis Image Analysis (Onimagin Technologies SCA, Cordoba, Spain) (26). Total tube lengths were counted in five blind fields per condition, averaged, and then compared across the experimental groups.

### Enzyme-Linked Immunosorbent Assay (ELISA)

HUVECs were seeded into 6-well plates with at the density of 150,000 cells/well. The following day, cells were incubated for further 48 h with MPs (20 μg/ml) derived from untreated HUVECs (CTL MPs) and those treated with TG or TG + PBA. Untreated cells (no MPs treatment) were used as a negative control. Culture media were then collected from all conditions and interleukin (IL)-6 concentration (pg/ml) was determined using a commercial ELISA colorimetric kit following the manufacturer's instructions (R&D Systems, Minneapolis, USA). Samples and IL-6 standards were loaded on a pre-coated microplate. Absorbance was then read at 450 nm with a reference wavelength correction set to 570 nm using a H1 synergy microplate reader (BioTek Instruments, Winooski, USA).

### Indirect NO Quantification (Griess Assay)

HUVECs were seeded into 6-well plates with at the density of 150,000 cells/well. The following day, cells were incubated for further 48 h with MPs (20 μg/ml) derived from untreated HUVECs (CTL MPs) and those treated with TG or TG + PBA. Untreated cells (no MPs treatment) were used as a negative control. Then, the concentration of NO stable metabolites, nitrite and nitrate, were quantified using Griess assay following the manufacturer's recommendations (R&D Systems, Minneapolis, USA). Nitrite and nitrate concentration in culture media of cells reflect the levels of NO produced by cells. After treatments, culture media were collected, centrifuged to remove any debris. Nitrates in the samples were converted to nitrites by applying nitrate reductase. Finally, nitrite concentrations were determined by following the supplier instructions using a standard curve with nitrite. The concentration of the nitrite formed was proportional to the amount of NO produced by the cells when measured at 540 nm with wavelength correction at 690 nm using H1 synergy microplate reader (BioTek Instruments, Winooski, USA).

## Statistical Analysis

Results are expressed as mean ± SEM, and *n* represents the number of biological repeats. Statistical analyses were performed with GraphPad Prism® 7.01e software for MAC using either one-way ANOVA or two-way ANOVA followed by Tukey's or Bonferroni multiple comparison *post hoc* tests, respectively. A two-tailed *P* ≤ 0.05 was considered as statistically significant.

## Results

### ER Stress Induction Did Not Cause Quantitative Differences in MPs Generated

**Figure 1:**
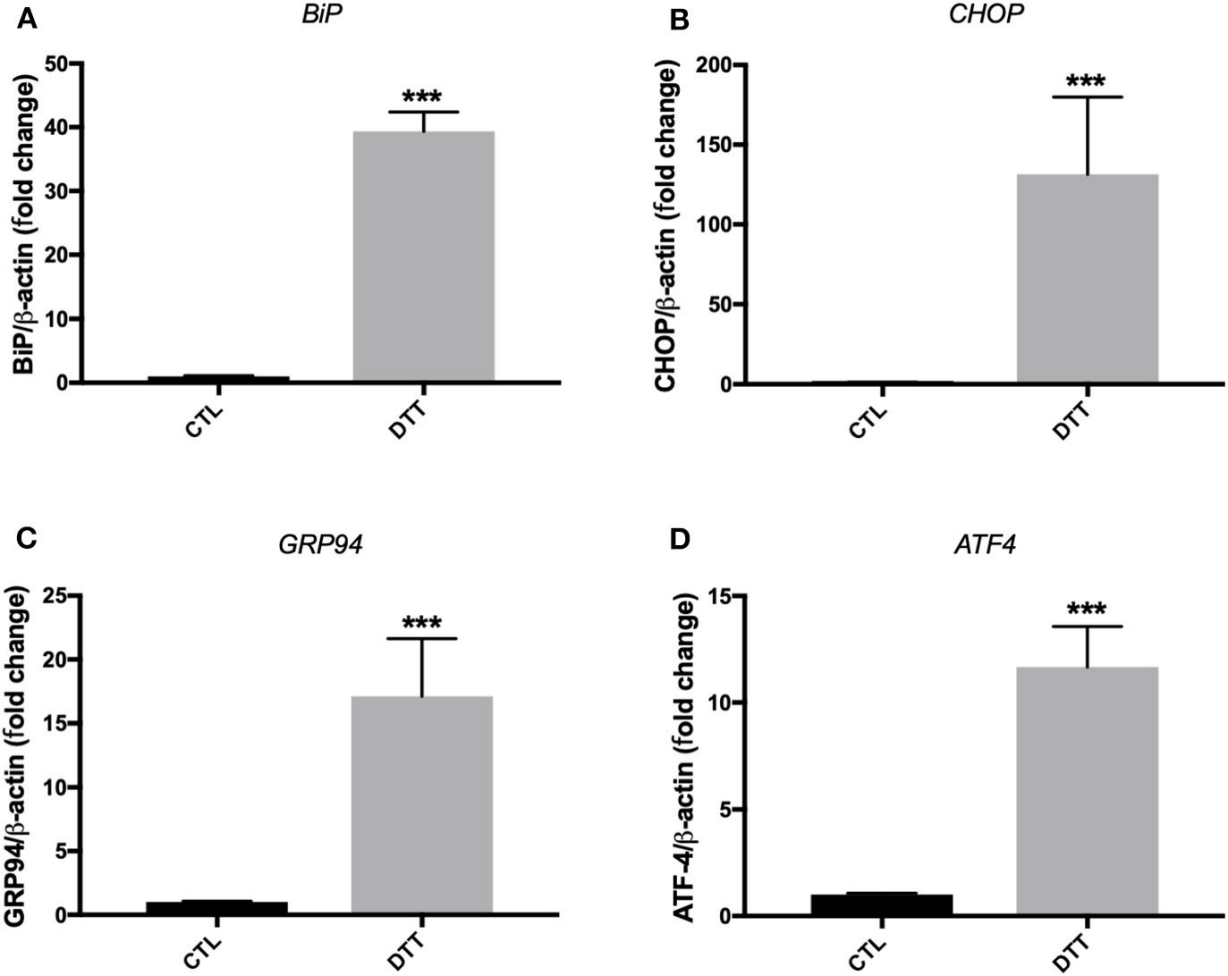
ER stress induction with pharmacological inducer of ER stress, DTT, in EA.hy926 endothelial cells. Relative mRNA expression by RT-qPCR of ER stress markers: *BiP* **(A)**, *CHOP* **(B)**, *GRP94* **(C)**, and *ATF-4* **(D)** normalized against housekeeping gene β*-actin*. Dithiothreitol (DTT) was used in concentration of 2 mM for 24 h to induce ER stress (*n* = 3–4). Data are presented as mean ± SEM. Data were analyzed by one-way ANOVA and Tukey's multiple comparison test. ***P < 0.001 vs. control (CTL).

**Figure 2:**
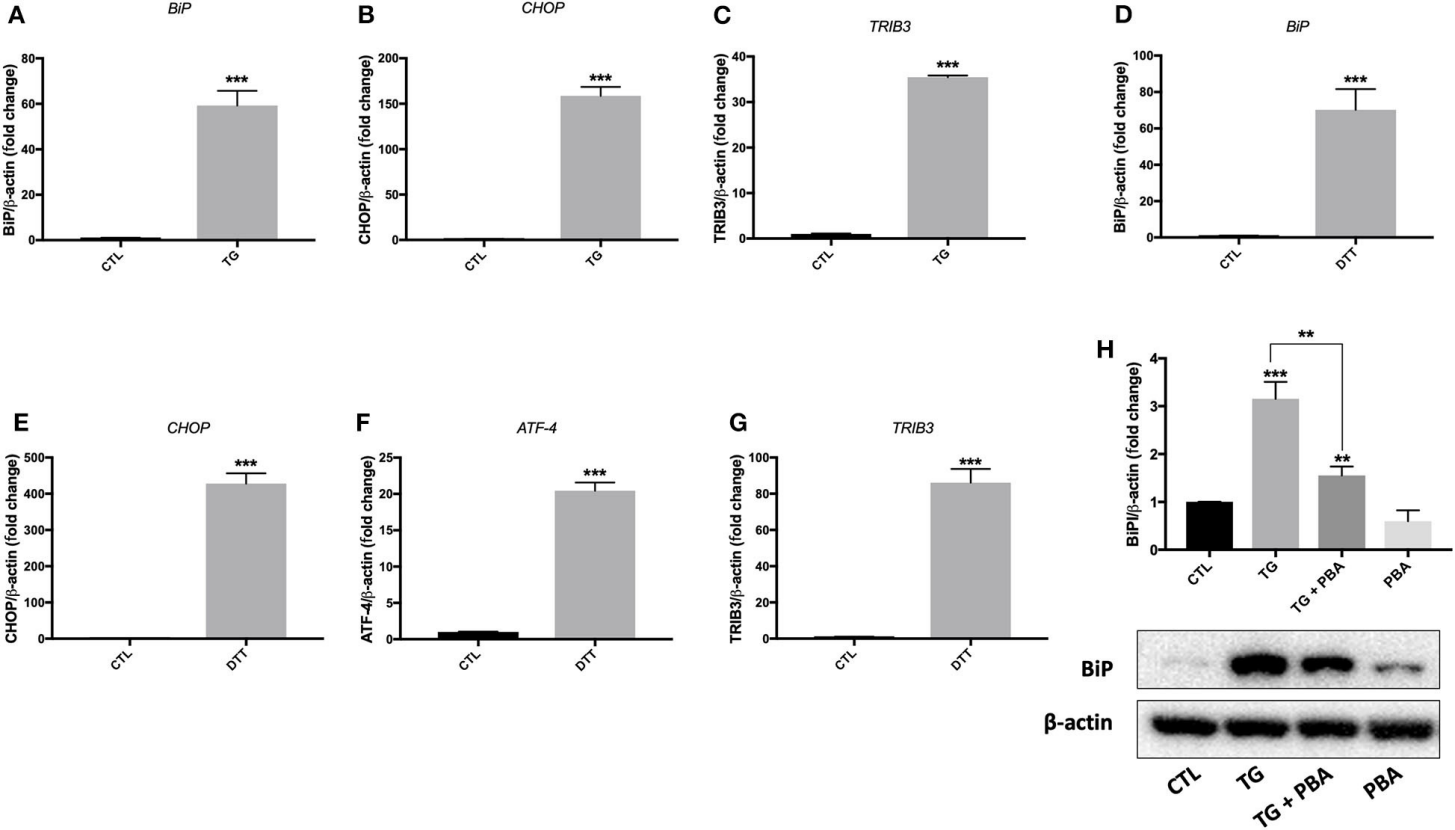
ER stress was activated by TG and DTT in HUVECs. Relative mRNA expression by qPCR of ER stress markers: *BiP* **(A)**, *CHOP* **(B)**, and *TRIB3* **(C)** in HUVECs treated with thapsigargin (TG; 300 nM, 24 h) and *BiP* **(D)**, *CHOP* **(E)**, *ATF-4* **(F)** and *TRIB3* **(G)** in HUVECs treated with Dithiothreitol (DTT; 2M, 24 h) normalized against housekeeping gene β*-actin* (*n* = 3–4). **(H)**, Western blot of protein expression of BiP in HUVECs treated with thapsigargin (TG; 300 nM, 24 h) in the presence or absence of PBA (10 mM). Bars represent pooled densitometry data normalized to total amount of β-actin loading control expressed as fold change compared to control (CTL) (*n* = 4 per group). Data are presented as mean ± SEM. Data were analyzed by one-way ANOVA followed with Tukey's multiple comparison test. **P < 0.01, ***P < 0.001 vs. control (CTL) or vs. indicated group.

Treatment of EA.hy926 endothelial cells with DTT (2 mM) for 24 h resulted in enhanced mRNA expression of ER stress target markers; *BiP, CHOP, GRP94 and ATF-4* (Figure 1). Treatment of HUVECs with TG (300 nM) for 24 h resulted in an increase in mRNA expression of *BiP, CHOP* and *TRIB3* (Figures 2A–C), while their incubation with DTT (2 mM; 24 h) caused an increase in mRNA expression of *BiP, CHOP, ATF-4*, and *TRIB3* (Figures 2D–G), indicating the successful induction of ER stress in both cell lines. As shown in Figure 2H, the treatment of HUVECs with TG for 24 h caused an increase in protein expression of BiP (Figure 2H). Interestingly, pre-treatment of cells with PBA (10 mM) partially, but significantly, prevented the increase in BiP protein expression caused by TG (Figure 2H).

**Figure 3:**
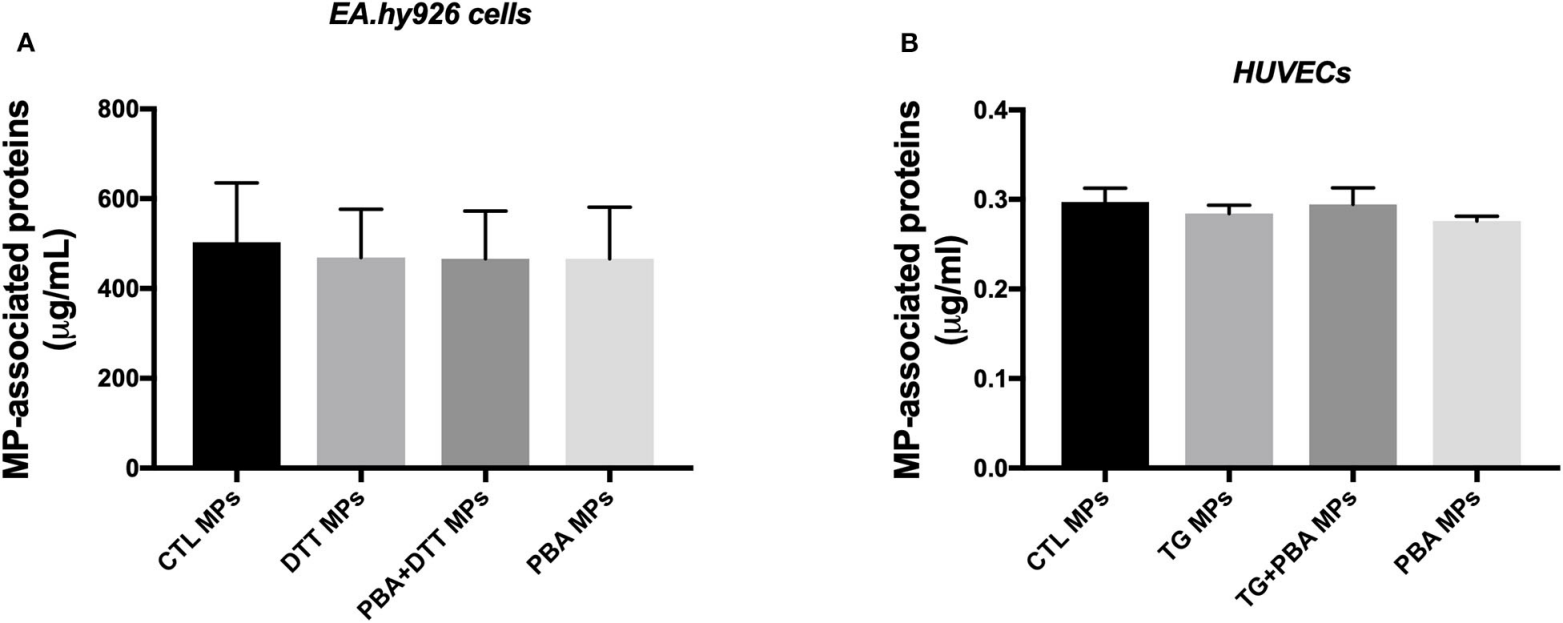
Quantification of MPs-associated proteins generated from endothelial cells. **(A)**, Concentration of total proteins (μg/ml) associated with MPs derived from EA.hy926 endothelial cells treated with DTT (DTT MPs), DTT + PBA (DTT + PBA MPs) and PBA (PBA MPs); MPs were collected after 24 h of treatment (*n* = 4 per group). **(B)**, Concentration of proteins (μg/ml) associated with MPs derived from HUVECs treated with TG (TG MPs) and TG + PBA (TG + PBA MPs) and PBA (PBA MPs); MPs were collected after 24 h of TG treatment (*n* = 9 per group). Data are presented as mean ± SEM and analyzed by one-way ANOVA followed by Tukey's multiple comparison test.

Analysis of MPs isolated from various conditions showed no differences in amount of proteins associated with MPs isolated from EA.hy926 cells treated with DTT in the presence or absence of PBA (Figure 3A) or from HUVECs incubated with TG in the in the presence or absence of PBA (Figure 3B). These data suggest that ER stress induction while caused shedding of MPs, it did not increase its release compared to control conditions.

### ER Stress-Generated MPs Activated ER Stress in a Vicious Circle in Both EA.hy926 Cells and HUVECs

**Figure 4:**
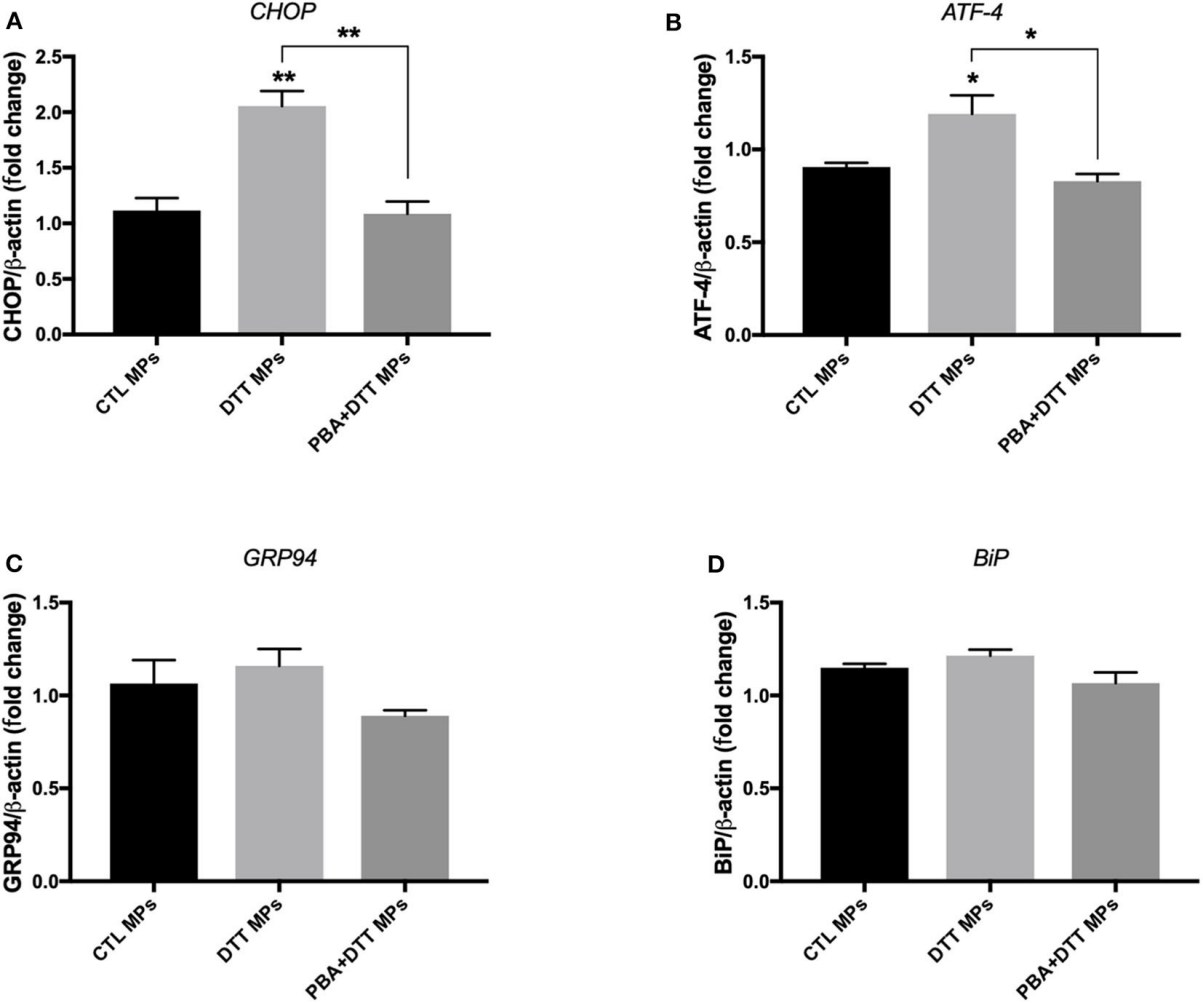
ER stress-generated MPs activated ER stress in EA.hy926 endothelial cells. Relative mRNA expression levels of ER stress markers of *BiP* **(A)**, *ATF-4* **(B)**, *GRP94* **(C)**, and *BiP* **(D)** in EA.hy926 endothelial cells after treatment with MPs (10 μg/ml for 24 h) normalized against housekeeping gene β*-actin* (*n* = 3 per group). Data are presented as mean ± SEM and were analyzed by one-way ANOVA followed with Tukey's multiple comparison test. *P < 0.05, **P < 0.01 vs. CTL MPs or vs. indicated groups.

EA.hy926 cells were treated with ER stress-generated MPs for 24 h, at MPs concentration of 10 μg/ml. As shown in Figure 4, treatment of cells with DTT-generated MPs caused an increase in mRNA expression of *CHOP* (Figure 4A) and *ATF-4* (Figure 4B), while no significant changes were observed for *GRP94* (Figure 4C) and *BiP* (Figure 4D) compared to control MPs, indicating activation of ER stress response. Interestingly, MPs generated from EA.hy926 cells stimulated with TG in presence of PBA (TG+PBA MPs) did not cause any changes in mRNA expression of ER stress markers studied compared to control MPs (CTL MPs) (Figure 4).

**Figure 5:**
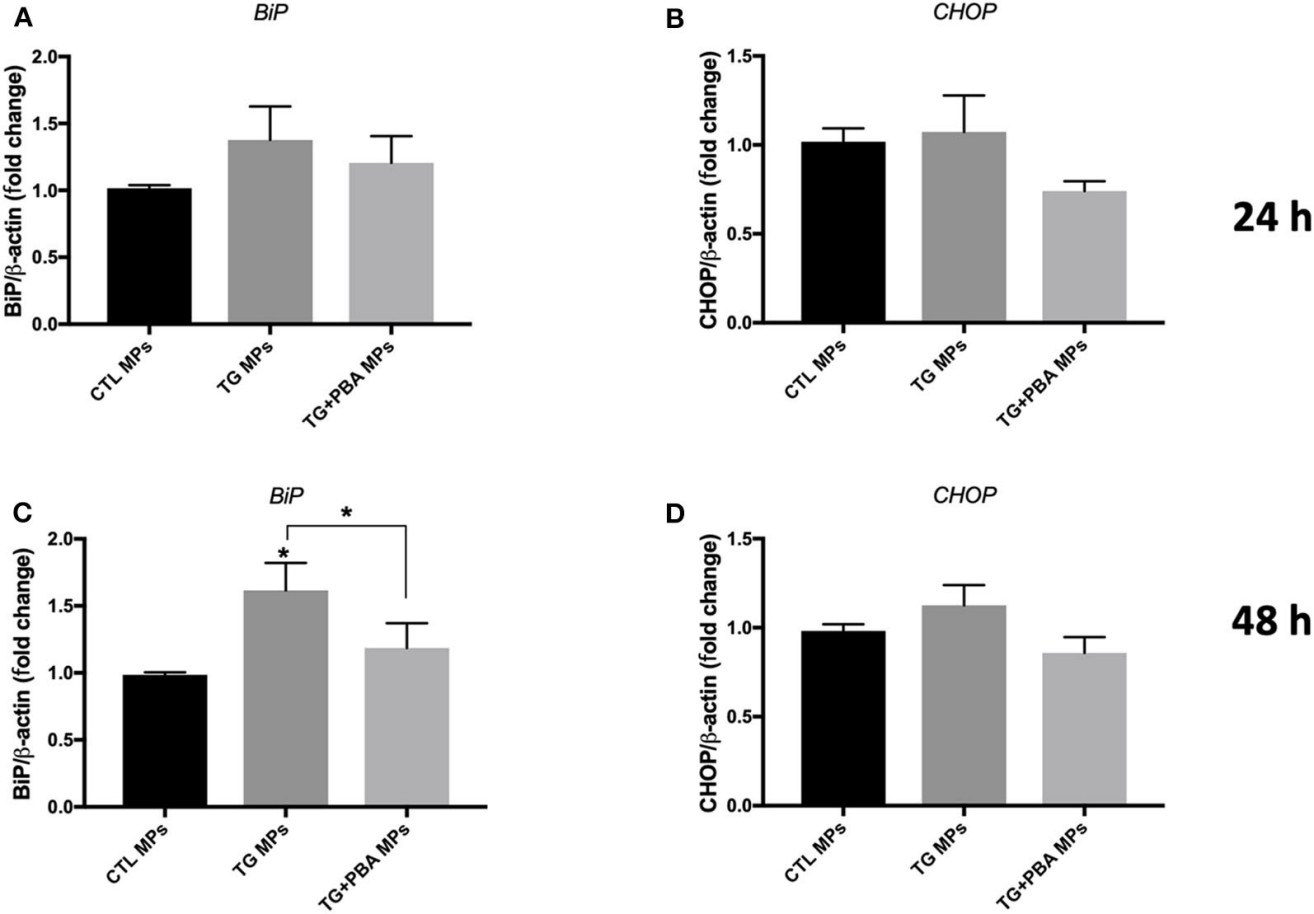
ER stress-generated MPs (10 μg/ml) activated ER stress in HUVECs. Relative mRNA expression levels of ER stress markers of *BiP* **(A,C)** and *CHOP* **(B,D)** in HUVECs treated with CTL MPs, TG MPs and TG + PBA MPs at a concentration of 10 μg/ml for 24 and 48 h (*n* = 4 for each group) normalized against house-keeping gene β*-actin*. Data are presented as mean ± SEM and were analyzed by one-way ANOVA followed with Tukey's multiple comparison test. *P < 0.05 vs. CTL MPs or vs. indicated groups.

**Figure 6:**
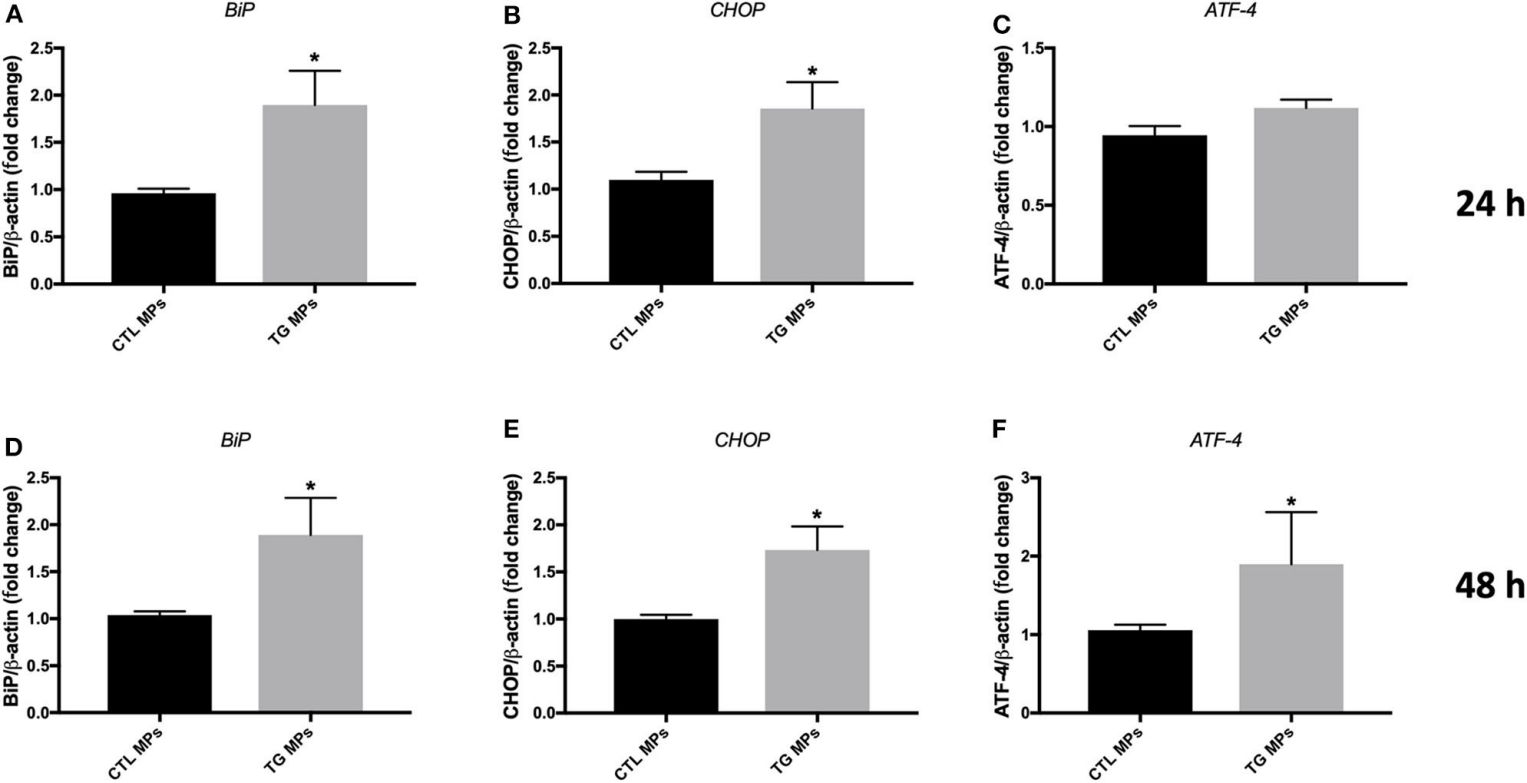
ER stress-generated MPs (20 μg/ml) activated ER stress in HUVECs. Relative mRNA expression levels of ER stress markers *BiP* **(A,D)**, *CHOP* **(B,E)**, and *ATF-4* **(C,F)** in HUVECs treated with CTL MPs, TG MPs at a concentration of 20 μg/ml for 24 and 48 h (*n* = 4 in each group), normalized against housekeeping gene β-actin. Data are presented as mean ± SEM and were analyzed by one-way ANOVA followed with Tukey's multiple comparison test. *P < 0.05 vs. CTL MPs.

On the other hand, the treatment of HUVECs with TG-generated MPs at the concentration of 10 μg/ml caused an increase in mRNA expression of *BiP* (Figure 5C) and a trend for increase for *CHOP* (Figure 5D) after 48 h incubation, while incubation of cells for 24 h (Figures 5A,B) did not show differences compared to control MPs. MPs generated from HUVECs stimulated with TG in presence of PBA (TG + PBA MPs) did not cause any changes in mRNA expression of ER stress markers studied compared to control MPs (CTL MPs) (Figure 5). The stimulation of HUVECs with TG-generated MPs at a higher concentration of 20 μg/ml for both 24 and 48 h also caused an increase in mRNA expression of *BiP, ATF-4* and *CHOP* compared to control MPs at both time points (Figure 6).

Altogether, these data indicate that MPs generated under ER stress conditions can induce and self-perpetuate ER stress response in naïve endothelial cells, suggesting a vicious circle between shedding of MPs and activation of ER stress response.

### ER Stress-Generated MPs Impaired Tube-Like Structures Formation in HUVECs

**Figure 7:**
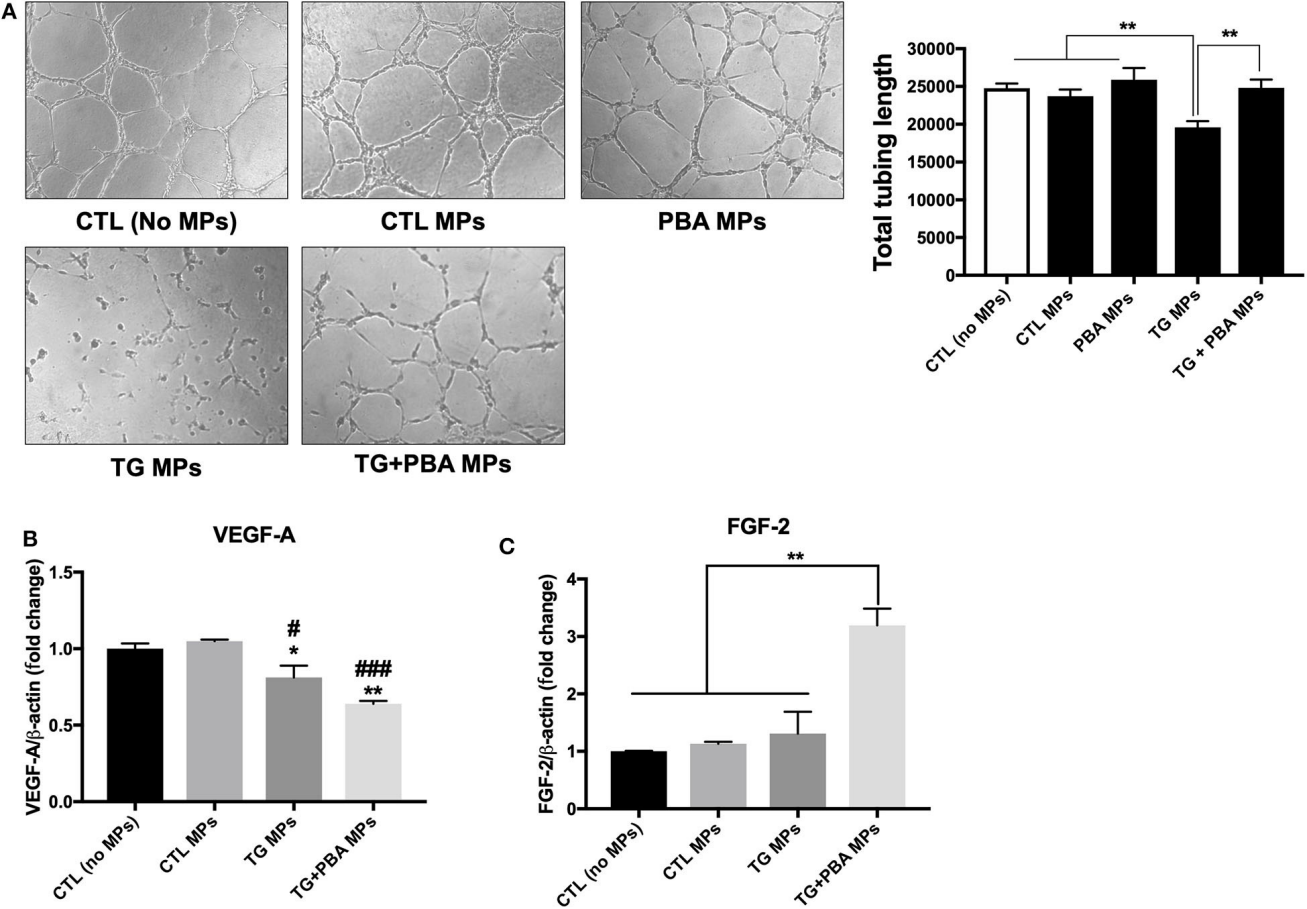
ER stress-generated MPs impaired angiogenic capacity in HUVECs. **(A)**, HUVECs were incubated in complete M200 medium (CTL or no MPs), or in complete culture medium after treatment with CTL MPs, TG MPs, TG+PBA MPs or PBA MPs (20 μg/ml for 48 h). Angiogenic capacity of HUVECs was assessed by Matrigel-based tube formation assay. Images are representative of three independent experiments; images were taken after 4 h. To quantify angiogenesis, the length of tubes formed was counted in five random fields for each well using WimTube software from Wimasis Image Analysis. Bars represent the pooled data expressed as the total length of tubes formed in each condition. **(B,C)**, relative mRNA expression levels of angiogenic factors *VEGF-A* **(B)** and *FGF-2* **(C)** after treatment with MPs (20 μg/ml for 48 h), normalized against housekeeping gene β-actin (*n* = 3 per group). Data are presented as mean ± SEM and were analyzed by one-way ANOVA followed with Tukey's multiple comparison test. *P < 0.05, **P < 0.01 vs. CTL (No MPs) or vs. indicated groups; ##*P* < 0.01, ###*P* < 0.001 vs. CTL MPs.

HUVECs were incubated with MPs generated under ER stress conditions (TG MPs) for 48 h before collecting the cells and growing them on Matrigel matrix for further 4 h to allow formation of tube-like structures, a marker of angiogenic capacity. As shown in Figure 7A, TG-generated MPs were able to impair tube-like structures formation on a Matrigel matrix, compared to control (no MPs), CTL MPs or PBA MPs (Figure 7A). Of note, MPs generated from cells treated with TG in presence of PBA (TG + PBA MPs) did not affect angiogenic capacity of HUVECs compared to control (no MPs), CTL MPs or PBA MPs (Figure 7A). These data support that ER stress-generated MPs can cause endothelial dysfunction as evidenced by reduced capacity to form tube-like structures when grown on a Matrigel three-dimensional matrix.

To further harness the effects of MPs on angiogenic capacity, we evaluated mRNA expression of 2 pro-angiogenic factors, Vascular endothelial growth factor A (*VEGF-A*) and basic fibroblast growth factor (*FGF-*β or *FGF-2*). As shown in Figure 7B, TG MPs and TG + PBA MPs reduced mRNA expression of *VEGF-A* compared to control (no MPs) and CTL MPs; however, this decrease was compensated by an increase in *FGF-2* mRNA expression only in the group of cells treated with TG + PBA MPs (Figure 7C).

**Figure 8:**
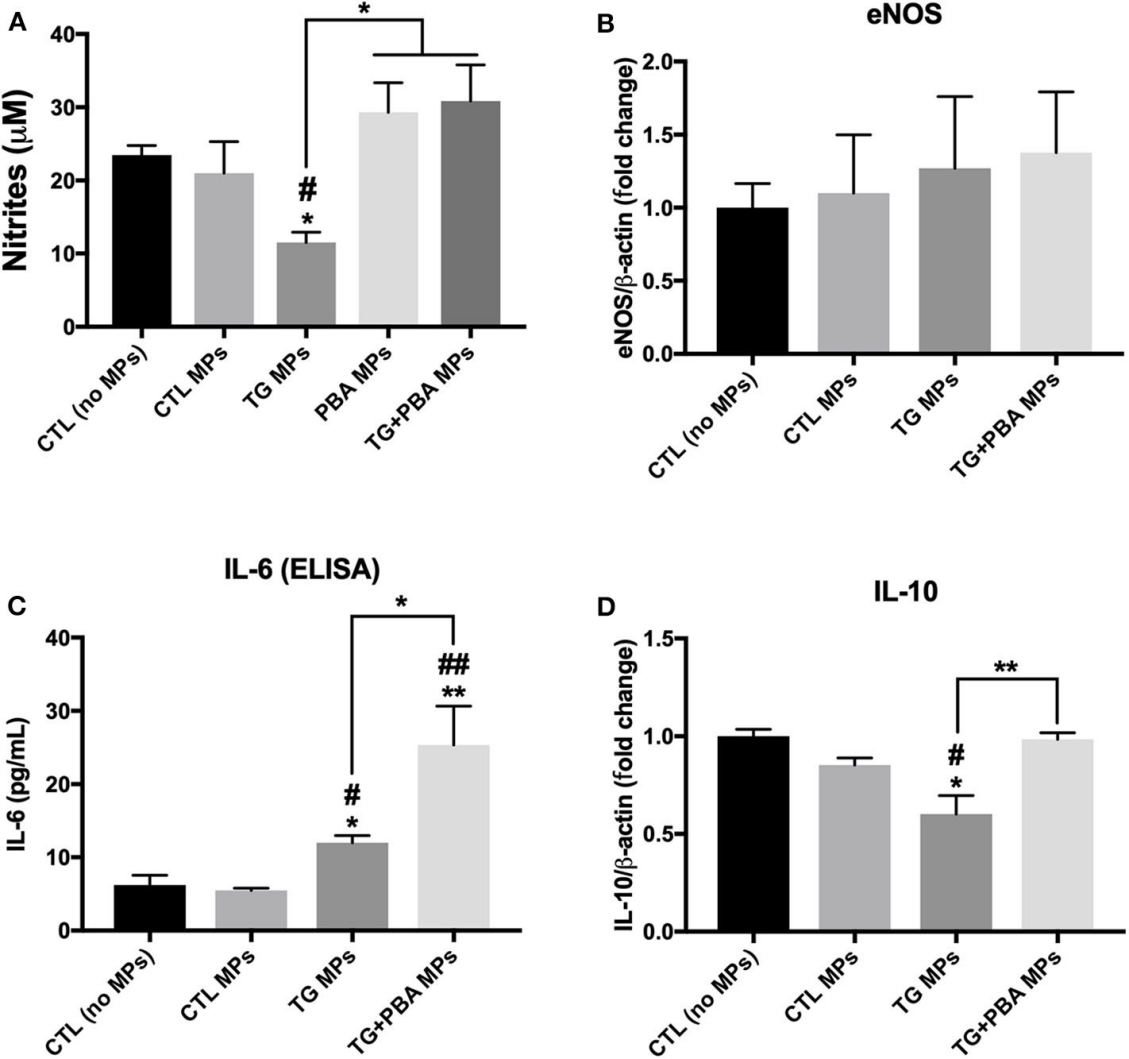
Effects of TG-generated MPs on inflammatory cytokines and NO release. **(A)**, Nitrites concentration (μM), indicator of NO levels, in culture media from untreated HUVECs (CTL, no MPs) and cells treated with CTL MPs, TG MPs, TG+PBA MPs or PBA MPs at a concentration of 20 μg/ml for 48 h (*n* = 6 per group). **(B)**, Relative mRNA expression of *eNOS* in HUVECs after treatment with MPs (20 μg/ml for 48 h) normalized against housekeeping gene β*-actin* (*n* = 3 per group). **(C)**, IL-6 concentrations (pg/ml) determined by ELISA in culture media from control (No MPs) HUVECs and those treated with CTL MPs, TG MPs, TG + PBA MPs or PBA MPs at a concentration of 20 μg/ml for 48 h (*n* = 3). **(D)**, Relative mRNA expression levels of *IL-10* in HUVECs after treatment with MPs (20 μg/ml for 48 h) normalized against housekeeping gene β*-actin* (*n* = 3 per group). Data are presented as mean ± SEM. One-way ANOVA test was used for analysis and Tukey's multiple comparison test was used as a *post-hoc* test. *P < 0.05, **P < 0.01 vs. control (No MPs) or vs. indicated groups; #*P* < 0.05, ##*P* < 0.01 vs. CTL MPs.

Given the importance of NO in the regulation of multiple functions of endothelial cells including angiogenesis, the levels of NO release were assessed in HUVECs exposed to MPs using the Griess assay. As shown in Figure 8A, treatment of cells with TG MPs (20 μg/ml for 48 h) caused a significant reduction in the concentration of nitrites while cells incubated with MPs generated from HUVECs treated with PBA alone or TG+PBA did not cause such a reduction (Figure 8A). This effect was not associated with any changes in mRNA expression of *eNOS* (Figure 8B).

Endothelial cell dysfunction is closely associated with cellular inflammation. As shown in Figure 8C, TG MPs caused an increase in the release of inflammatory cytokine IL-6 compared to CTL MPs and control (no MPs) groups. Simultaneously, cells treated with TG MPs showed a decrease in mRNA expression of anti-inflammatory cytokine *IL-10* while such decrease was not observed in cells treated with TG+PBA MPs (Figure 8D).

### ER Stress-Generated MPs Did Not Affect Cell Survival in HUVECs

**Figure 9:**
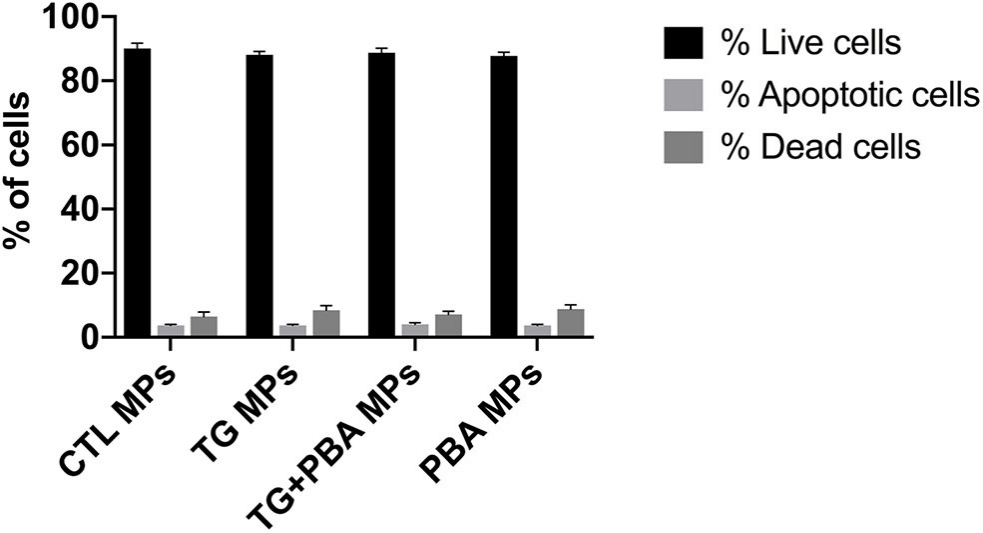
Apoptosis in HUVECs treated with ER stress-generated MPs. Assessment of apoptosis in HUVECs treated with CTL MPs, TG MPs, TG + PBA MPs or PBA MPs at a concentration of 20 μg/ml for 48 h (*n* = 3). Apoptosis was analyzed by Tali® Image-based Cytometry. Bars represent pooled data for live, apoptotic and dead cells expressed as percentage of total number of cells. Data are presented as mean ± SEM. Two-way ANOVA test was used to analyze data followed with Bonferroni multiple comparison test.

Apoptosis is an important molecular mechanism underpinning ER stress-mediated endothelial dysfunction and impaired angiogenic capacity (15, 18). We have therefore assessed the impact of ER stress activation by TG-generated MPs on cell survival of HUVECs. Cells were treated for 48 h with 20 μg/ml MPs, and apoptosis was then detected by Tali image-based cytometry. As shown in Figure 9, HUVECs treated with TG-generated MPs exhibited only 5% of apoptotic cells showing no statistical difference compared to control MPs-treated cells or all other treatment groups. These data indicate that impaired angiogenic capacity caused by TG-generated MPs was independent of an effect on cell survival.

### Differential Effects Between TG and TG-generated MPs on the Regulation of Inflammatory and Survival Signaling Pathways and Autophagy

To further harness the intercellular signaling pathways associated with the effects of TG-generated MPs on endothelial cells, we have compared the effects of TG and TG-generated MPs on the activation of key cellular pathways associated with cell survival and apoptosis and known to be activated during ER stress response in addition to autophagy.

**Figure 10:**
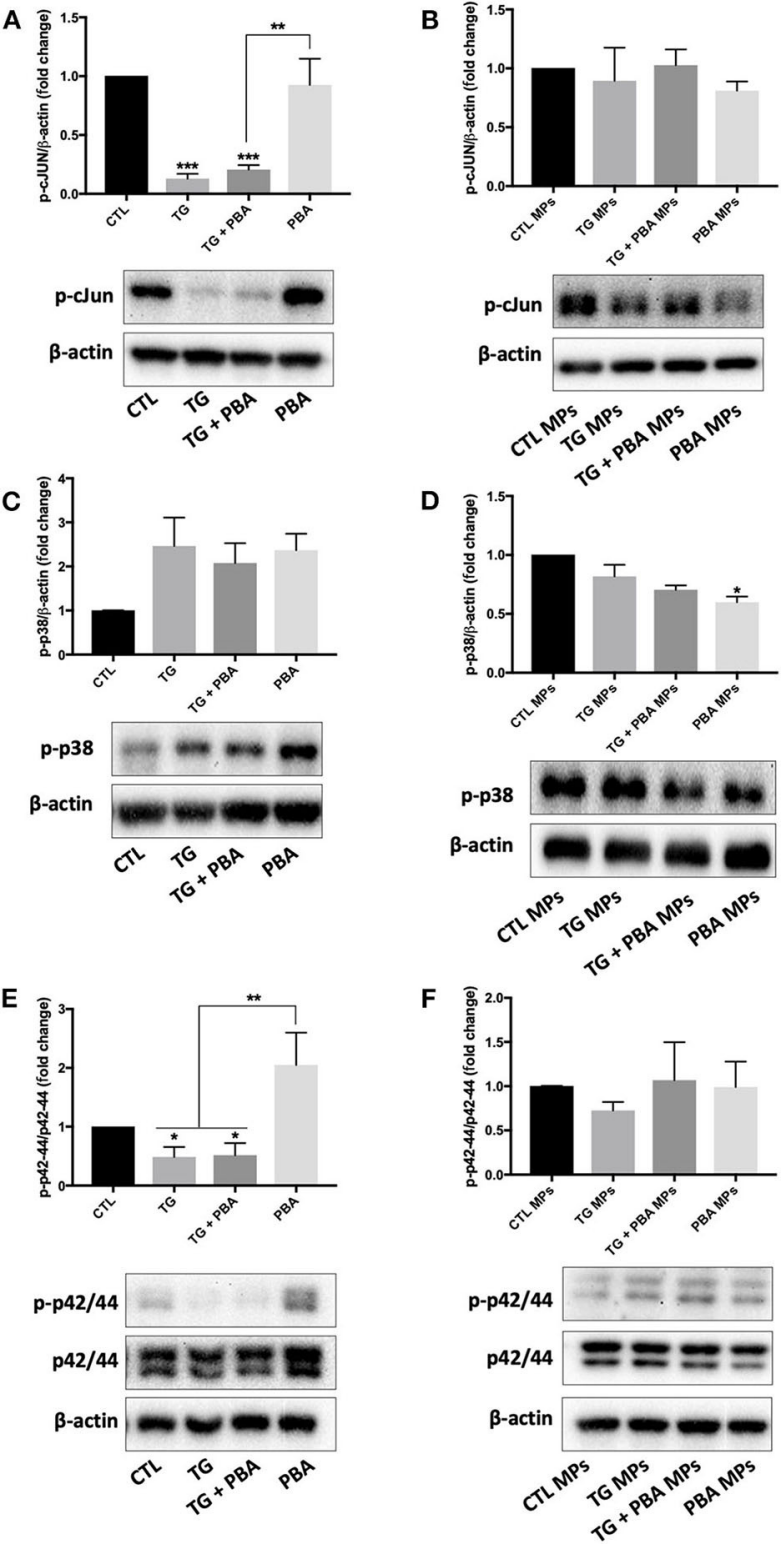
Differential effects between TG and TG-generated MPs on the regulation of inflammatory and survival signaling pathways. Western blot analysis of protein expression of ER p-cJUN **(A,B)**, p-p38 **(C,D)**, and p-42/44 **(E,F)** in HUVECs treated with TG (300 nM, 24 h) in the presence or absence of PBA (10 mM) or treated with CTL MPs, TG MPs, TG + PBA MPs or PBA MPs at a concentration of 20 μg/ml for 48 h (*n* = 3–6 per group). Representative images from more than three independent experiments are shown. Bars represent pooled densitometry data normalized to total amount of β-actin loading control or total p42/44 expressed as fold change compared to control (CTL or CTL MPs) (*n* = 4 per group). Data are presented as mean ± SEM. One-way ANOVA test was used for analysis and Tukey's multiple comparison test was used as a *post-hoc* test. *P < 0.05, **P < 0.01, ***P < 0.001 vs. CTL or CTL MPs or vs. indicated groups.

As shown in Figure 10A, the treatment of HUVECs with TG caused a decrease in the phosphorylation of c-JUN, an indicator of the activation of JNK pathway that was not rescued by PBA; however, the treatment of cells with TG-generated MPs did not affect p-cJUN expression (Figure 10B). On the other hand, while TG caused a trend to increase in the phosphorylation of p38 MAPK (Figure 10C), TG-generated MPs showed a trend for decrease (Figure 10D). With regards to the activation of proliferative p42/44 MAPK pathway, TG treatment caused a significant reduction in phosphorylation and hence activation of p42/44 MAPK, an effect that was not prevented by incubation of cells with PBA (Figure 10E). However, the treatment of HUVECs with TG-generated MPs did not affect the activation of p42/44 MAPK signaling response (Figure 10F).

**Figure 11:**
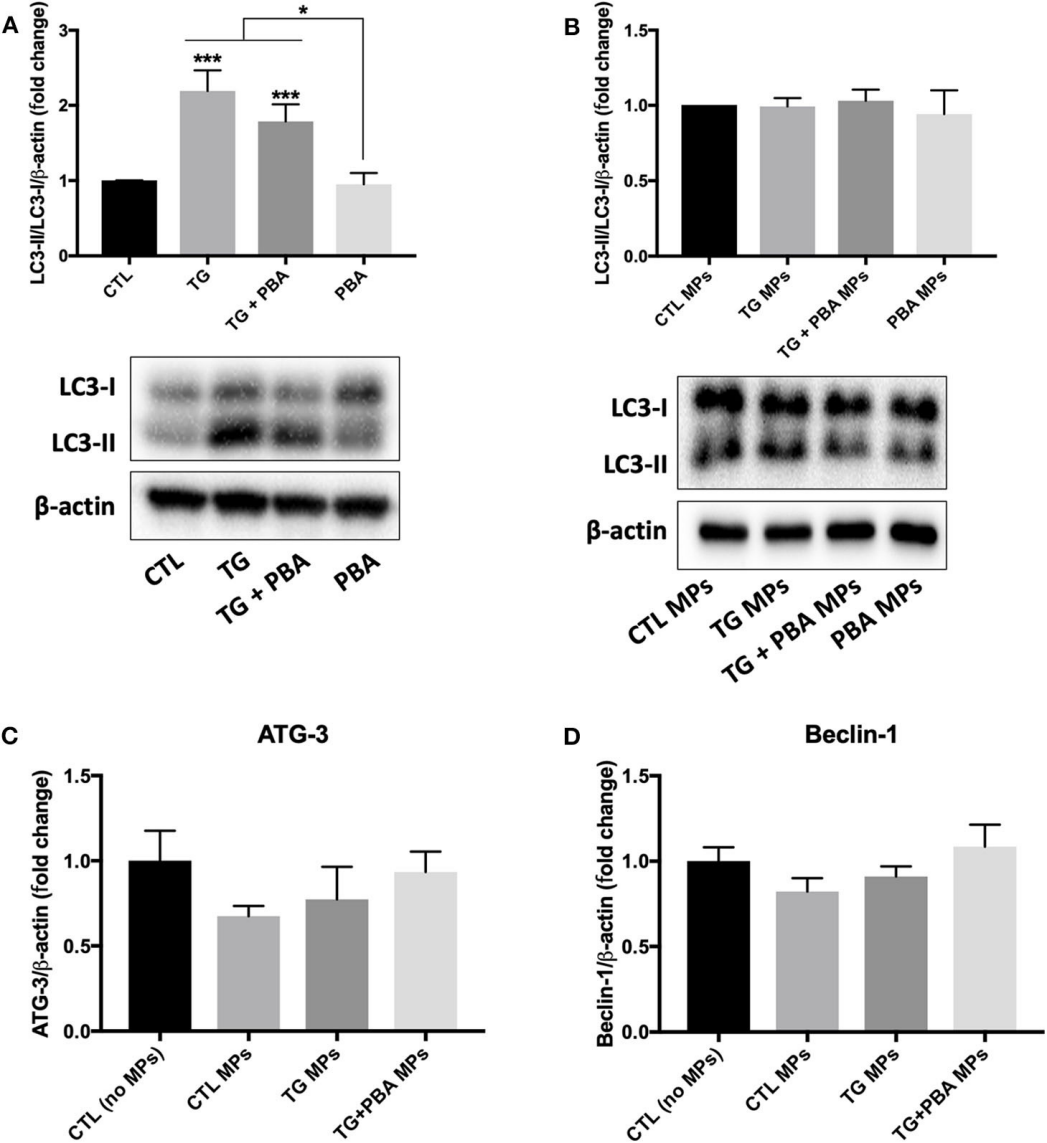
Differential effects between TG and TG-generated MPs on autophagy activation. **(A,B)**, Western blot analysis of autophagy marker (LC3 I/II) in HUVECs treated with TG (300 nM, 24 h) in the presence or absence of PBA (10 mM) **(A)** or treated with CTL MPs, TG MPs, TG+PBA MPs or PBA MPs at a concentration of 20 μg/ml for 48 h **(B)** (*n* = 3–6 per group). Bars represent autophagic rate expressed as the ratio of densitometry signal for LC3-II normalized to LC3-I and to total amount of β-actin loading control expressed as fold change compared to control (CTL or CTL MPs) (*n* = 4–6 per group). **(C,D)**, relative mRNA expression levels of *ATG-3* **(C)** and *Beclin-1* **(D)** after treatment with MPs (20 μg/ml for 48 h) normalized against housekeeping gene β*-actin* (*n* = 3 per group). Data are presented as mean ± SEM. One-way ANOVA test was used for analysis and Tukey's multiple comparison test was used as a *post-hoc* test. *P < 0.05, ***P < 0.001 vs. CTL or vs. indicated groups.

As shown in Figure 11, there was a differential effect of TG and TG-generated MPs on the activation of autophagy. TG caused a significant increase in autophagic rate as indicated by the increase in the ratio between LC3-II and LC3-I (indicative of the conversion of LC3-I into LC3-II) (Figure 11A). However, the treatment of HUVECs with TG-generated MPs did not affect the ratio between LC3-II and LC3-I (Figure 11B). The analysis of mRNA expression of *Beclin-1* (Figure 11C) and *autophagy related (ATG)-3* (Figure 11D), two additional makers of autophagy, revealed that treatment of HUVECs with MPs did not affect their expression levels corroborating protein expression results of LC3-I/II, indicating the absence of autophagy induction, at the concentrations and time exposure used for MPs.

Altogether, these data indicate that ER stress-generated MPs caused endothelial dysfunction in a mechanism that is independent from cell survival and autophagy despite their capacity to induce and hence self-perpetuate ER stress response.

## Discussion

Understanding the molecular mechanisms underlying endothelial dysfunction, an important contributing factor to the initiation of atherosclerosis that progresses to vascular complications, is warranted to identify targets for therapy and/or prevention. The underlying mechanisms of endothelial dysfunction are not fully clear; however, ER stress activation was shown to be implicated *in vitro* (18, 27) and *in vivo* (28, 29) in impaired endothelial cell functions. MPs have strongly emerged as biomarkers and mediators of endothelial dysfunction in diabetes and very seldom evidence is showing the implication of ER stress activation in MP-induced endothelial dysfunction (20, 30, 31).

In this project, we investigated the effects of MPs generated from endothelial cells under ER stress conditions on endothelial cell function. We confirmed here, for the first time, that subjecting endothelial cells to ER stress caused shedding of MPs and that ER stress-generated MPs were capable themselves of causing activation of ER stress in healthy endothelial cells in a vicious circle between ER stress activation and production of MPs. Previously, only one study reported that MPs from metabolic syndrome patients and apoptotic T-lymphocytes, induced endothelial dysfunction, partly, through the activation of ER stress response (20). Here, we showed that ER stress-generated MPs from endothelial cells, impaired angiogenic capacity *in vitro*, a feature of endothelial dysfunction, independently of cell survival. Our study contributes to the general understanding of the deleterious effects mediated by MPs. MPs exert their effects through their biological content. Therefore, future studies of the composition of MPs and determining the exact role of the biological materials in MPs-mediated pathological effects will aid further in identifying more specific therapeutic targets.

In our study, ER stress induction by TG or DTT for 24 h did not increase MPs production from endothelial cells compared to control. Our study is the first to report the effects of pharmacological ER stress induction on MP release in endothelial cells. In another cell type, smooth muscle cells (SMCs), it has been reported that shear stress-mediated ER stress caused a significant increase in the release of MPs compared to control (32). Unlike our study, Jia et al. (32) observed an increase in MPs shed from SMCs subjected to shear stress-mediated ER stress. The differences in cell types used (HUVECs vs. SMCs) and ER stress stimuli (shear stress for 48 h vs. DTT or TG) may explain differences in the impact of ER stress activation on MP shedding in the two cell types. Importantly, in our study, we observed that ER stress-generated MPs were different in relation to their effects exerted on normal endothelial cells compared to the control MPs, indicating that although quantitatively there were no differences, ER stress-shed MPs were qualitatively different as they carried out deleterious biological messages against endothelial function as evidenced by impaired angiogenic capacity.

In our study, we reported, for the first time, that there is a vicious circle between ER stress activation in endothelial cells and generation of deleterious MPs that can themselves activate ER stress in naïve endothelial cells. Only one study was found in the literature showing the implication of ER stress in MPs-induced endothelial dysfunction (20); however, authors did not investigate the impact of ER stress on MP release. Our novel findings here harness the link between ER stress and MPs and improve our current understanding of cellular responses underpinning the release and action of MPs in disease. As noted in Figures 4–6, there were differences in the potency of ER stress induction caused by TG and DTT-generated MPs in EA.hy926 cells and HUVECs. DTT-generated MPs, used at the concentration of 10 μg/ml (24 h), were more potent in causing ER stress in EA.hy926 cells compared to the effects of TG-generated MPs in HUVECs used at the same concentration and incubation duration (24 h). This differential response of ER stress-generated MPs was observed although both TG and DTT were very potent in inducing ER stress in EA.hy926 cells and HUVECs (Figure 1). This can be explained by the differences in the mechanisms of ER stress activation by TG (intracellular calcium stores depletion) and DTT (reducing inter- and intra-protein disulfide bonds) that led to the shedding of MPs in addition to differences in the nature of cells where HUVECs are primary cells and EA.hy926 are immortalized cells. One limitation, however, to be highlighted here is that the effects of TG- and DTT-generated MPs were not tested in both cell types. We further support here that the qualitative content, which reflects the nature of the biological messages transferred to target cells, is more important than quantity of MPs in the initiation and maintenance of disease processes.

Angiogenesis involves cell migration and proliferation. Treatment of naïve HUVECs with ER stress-generated MPs impaired their capacity to form tube-like structures on a three-dimensional Matrigel matrix but did not affect apoptosis or protein expression of major pro-apoptotic (p-cJun) and proliferative (p-p38 and p-p42/44 MAPK) signals. This may indicate that impaired angiogenic effect is mediated through the inhibition of cell migration but not proliferation and cell survival. Future studies are warranted to determine the exact role of ER stress-generated MPs on endothelial cell migration. The reduction in angiogenic capacity caused by TG MPs was associated with a decrease in mRNA expression of *VEGF-A* and *FGF-2*, which may contribute to the deleterious effect of TG MPs on angiogenesis, while cells exposed to TG + PBA MPs showed an enhanced mRNA expression of *FGF-2*. Importantly, the treatment of HUVECs with TG MPs significantly reduced NO release unlike cells exposed to PBA MPs or TG + PBA MPs. Reduced NO bioavailability is a major contributor to impaired angiogenesis (15, 18).

It was noted that the mother cells from which MPs were derived showed differential effects of TG on the activation of antioxidants and pro-apoptotic and survival pathways studied in addition to autophagy, when compared to cells treated with ER stress-generated MPs. Of note, in contrast to TG-treated cells, where expression of p-cJUN was lower, TG-generated MPs did not affect the activation of JNK pathway. Similarly, TG-treated cells showed a reduced activation of the proliferative pathway p42/44 MAPK, while TG-generated MPs did not affect this signaling response. Furthermore, unlike TG-treated cells, the autophagic ratio (LC3-II/LC3-I) was not affected by treatment with ER stress-generated MPs. This was further confirmed by the absence of changes in mRNA expression of 2 other markers of autophagy, *Beclin-1* and *ATG-3*, in cells treated with MPs. ER stress activates autophagy under the control of IRE-1α which mediates the activation of MAPK leading at the end to the activation of autophagy (33). These data indicate a dissociation between the implications of the pharmacological- or MPs- induction of ER stress in endothelial cells. The effects of pharmacological ER stress appear to be driven by an impairment in cell survival, while ER stress-generated MPs mediate their effects on endothelial cells via a subtler regulation of intracellular signaling responses that are independent from apoptosis and cell survival. It is important to note that ER stress-generated MPs were used up to the concentration of 20 μg/ml and up to 48 h; longer exposure and/or higher concentration may affect cell survival and viability of endothelial cells; however, higher concentrations may not be relevant patho-physiologically because we have reported previously that circulating endothelial MPs metabolic syndrome patients for example represent only 2–3% of total MPs (10). Maamoun et al. (18) found that high glucose concentrations impaired angiogenesis in HUVECs through ER stress-mediated cell death in a mechanism involving proapoptotic pathway JNK that is closely linked to sustained ER stress. Moreover, they found that treatment of endothelial cells with PBA reduced apoptosis indicating that endothelial cell death was mediated by ER stress (18). In our study, ER stress-generated MPs induced ER stress and impaired angiogenesis in endothelial cells without affecting apoptosis, autophagy or antioxidant production involving probably other mechanisms linked to ER stress such as oxidative stress (15). Of note, we observed that MPs generated from cells exposed to TG and those exposed to TG + PBA MPs increased release of inflammatory cytokine IL-6 as assessed by ELISA, which can contribute to the impairment of angiogenic capacity. Interestingly, mRNA expression of *IL-10*, a major anti-inflammatory cytokine, was reduced in cells treated with TG MPs, but preserved in cells incubated with TG+PBA MPs, which may contribute to counterbalancing the effects of increased IL-6 release in cells treated with TG+PBA MPs.

In conclusion, this study demonstrated that subjecting endothelial cells to ER stress resulted in the production of MPs, which were able to activate ER stress themselves in naïve endothelial cells in a vicious circle. TG-generated MPs, at the concentrations used, were able to reduce the capacity of HUVECs to form tube-like structures on a three-dimensional reconstituted basement membrane, without affecting autophagy or cell survival. This work enhances the general understanding of the deleterious effects carried out by MPs in medical conditions where ER stress is sustainably activated such as diabetes, obesity and metabolic syndrome. Our findings are clinically relevant as they will help in the process of identifying new therapeutic targets against MPs produced in conditions characterized by the activation of ER stress such as diabetes. MPs production in conditions with underlying ER stress were shown to initiate endothelial dysfunction. Therefore, MPs can be targeted by controlling their release from the cells, inhibiting their cellular uptake by the receipt cell or modulating their cargo content. For instance, several antidiabetics were reported to modulate the release of MPs in diabetic patients (34–36).

## Data Availability Statement

The raw data supporting the conclusions of this article will be made available by the authors, without undue reservation.

## Author Contributions

AA conceptualized and designed the study, administered the project and supervised the students and research associates, curated the data, and secured resources and funding. AO, HE-G, MP, SA, and MH performed experimental work. AA and AO performed the formal analysis, wrote the original draft of the manuscript, and prepared figures. HK, AZ, and TB contributed to analysis, discussion, reviewed, and edited the manuscript. All authors contributed to the article and approved the submitted version.

## Conflict of Interest

The authors declare that the research was conducted in the absence of any commercial or financial relationships that could be construed as a potential conflict of interest.
